# Cell-type specific methylation changes in the newborn child associated to obstetric pain relief

**DOI:** 10.1371/journal.pone.0308644

**Published:** 2024-09-19

**Authors:** Charles J. Tran, Thomas L. Campbell, Ralen H. Johnson, Lin Y. Xie, Christina M. Hultman, Edwin J. C. G. van den Oord, Karolina A. Aberg

**Affiliations:** 1 Center for Biomarker Research and Precision Medicine, Virginia Commonwealth University, Richmond, VA, United States of America; 2 Department of Medical Epidemiology and Biostatistics, Karolinska Institutet, Stockholm, Sweden; CHUK: Centre Hospitalier Universitaire de Kigali, RWANDA

## Abstract

Although it is widely known that various pharmaceuticals affect the methylome, the knowledge of the effects from anesthesia is limited, and nearly nonexistent regarding the effects of obstetric anesthesia on the newborn child. Using sequencing based-methylation data and a reference-based statistical deconvolution approach we performed methylome-wide association studies (MWAS) of neonatal whole blood, and for each cell-type specifically, to detect methylation variations that are associated with the pain relief administered to the mother during delivery. Significant findings were replicated in a different dataset and followed-up with gene ontology analysis to pinpoint biological functions of potential relevance to these neonatal methylation alterations. The MWAS analyses detected methylome-wide significant (q<0.1) alterations in the newborn for laughing gas in granulocytes (two CpGs, p<5.50x10^-9^, q = 0.067), and for pudendal block in monocytes (five CpGs across three loci, p<1.51 x10^-8^, q = 0.073). Suggestively significant findings (p<1.00x10^-6^) were detected for both treatments for bulk and all cell-types, and replication analyses showed consistent significant enrichment (odds ratios ranging 3.47–39.02; p<4.00×10^−4^) for each treatment, suggesting our results are robust. In contrast, we did not observe any overlap across treatments, suggesting that the treatments are associated with different alterations of the neonatal blood methylome. Gene ontology analyses of the replicating suggestively significant results indicated functions related to, for example, cell differentiation, intracellular membrane-bound organelles and calcium transport. In conclusion, for the first time, we investigated and detected effect of obstetric pain-relief on the blood methylome in the newborn child. The observed differences suggest that anesthetic treatment, such as laughing gas or pudendal block, may alter the neonatal methylome in a cell-type specific manner. Some of the observed alterations are part of gene ontology terms that previously have been suggested in relation to anesthetic treatment, supporting its potential role also in obstetric anesthesia.

## Introduction

Over the past decades, modern obstetric anesthesia, a sub-specialty of anesthesiology, has rapidly evolved with a plethora of clinical techniques utilized to provide mothers with pain relief during childbirth. Some of the most commonly used in hospital systems worldwide are laughing gas and pudendal block treatments [1, 2].

Laughing gas is a colloquial term for gaseous anesthetics. The most widely used laughing gas is nitrous oxide (N_2_O). Although various mechanisms of action may contribute to pain relief, N_2_O is generally believed to provide anesthesia by depressing neurotransmission of excitatory pathways in the central nervous system, likely via NMDA receptors, GABAa receptors and glutamate signaling [3, 4]. It has been shown that N_2_O crosses the placental barrier to the neonate when administered to the mother, implicating its ability to affect neonatal outcomes [5, 6]. Obstetric use of N_2_O is generally considered harmless to the neonate, but some studies have shown N_2_O to be potentially neurotoxic and genotoxic with adverse effects on hematologic and immunologic systems [7, 8]. For example, recent work suggested that N_2_O use during labor is a potential risk factor for infant vitamin B12 deficiency [9].

Pudendal blocks are commonly administered during the second stage of labor and are injected into the pudendal canal near the pudendal nerve. The mechanism of action for pudendal block anesthesia is believed to provide localized anesthesia by blocking voltage-gated ion (sodium, potassium, calcium) channels, which prevents depolarization for action potentials [10]. Local anesthetics have been shown to cross the placental barrier from mother to neonate [11, 12]. The transplacental transfer of anesthetic has been found in neonatal blood at a level of up to half of what mothers receive [13]. Similar to laughing gas the potential effect of pudendal block on the neonate is debated. Earlier studies found that pudendal blocks are not associated with any immediate effects on neonates [14–16]. However, some cases of neonatal methemoglobinemia have been reported following pudendal nerve block through transplacental mechanisms [17, 18].

Epigenetic modifications are essential for normal cell functioning and serve to provide phenotypic plasticity in response to various exogenous factors. One of the most frequently studied modifications is DNA methylation, where methyl groups bind to cytosine nucleotides at the C5 position. DNA methylation can, for example, inhibit transcription factor binding or promote gene repression, and ultimately regulate gene expression [19]. Aberrant methylation has been associated with a wide variety of human diseases [20–22] and life-style factors such as diet, pollutants, and pharmaceuticals, etc. [23, 24] have been reported as associated with variation in methylation patterns. Although it has been found that various pharmaceuticals affect the methylome [25], the information about the effects from anesthesia on methylation, in general, is limited, and to our knowledge, no studies exist regarding the effects of obstetric analgesia on the newborn child.

Here, we hypothesize that obstetric pain-relief administered to the mother during childbirth may alter the methylation profile of the child. To study this well, it is important to note that methylation patterns vary between cell-types within the same tissue [26]. Therefore, when analyzing bulk tissues, such as DNA extracted from whole blood that comprise many cell-types, significant variation may go undetected as the different cell-types may attenuate the effects of each other. To avoid this challenge, it is critical to perform analyses on a cell-type specific level.

In this study, we perform methylome-wide association studies (MWAS) of neonatal blood samples to detect potential methylation variations that are associated with the pain relief administered to the mother during delivery. In addition to performing these analyses in bulk tissue (i.e., whole blood), we use a statistical deconvolution approach that allows us to perform cell-type specific MWAS for the six most common cell-types in neonatal blood. Significant findings were replicated and followed up with gene ontology (GO) enrichment analysis to pinpoint biological functions of potential relevance to these neonatal methylation alterations.

## Materials and methods

### Participants

This study consists of participants enrolled in a larger Swedish schizophrenia study [27, 28]. All participants were born in Sweden from 1975 to 1989 and reported that their parents were of Swedish descent. Blood spots were collected by the Swedish hospital system via routine neonatal newborn screening, within 72 hours of birth. Phenotypic information, including information about sex, birth year, gestational age in days, and mother’s pain relief during child delivery was obtained from the Swedish National Registers. Blood spots and phenotypic information for this study were retrospectively accessed from November 2016 until November 2020. All participants were at least 18 years old and gave written informed consent to participate in the large Swedish study. All data was de-identified and coded thus, throughout and following the study, the authors did not have access to any information that could identify individual participants. The study was approved by institutional review boards in Sweden and at Virginia Commonwealth University (#HM20008809).

The total number of participants in this study is 222, which includes 77 individuals unexposed to obstetric anesthesia, 66 individuals exposed to laughing gas and 79 individuals whose mothers received pudendal block. The individuals exposed to each treatment are split into primary datasets including the individuals who exclusively received each of the studied anesthesia (laughing gas N = 35; pudendal block N = 48), and replication datasets (laughing gas N = 31; pudendal block N = 31), which includes participants who received the studied anesthesia in combination with other pain relief. No participants received the combination of laughing gas and pudendal block. Table 1 provides further descriptive information about the study participants.

**Table 1:** Descriptive information about the study participants.

	Untreated		Laughing Gas				Pudendal Block			
			Primary		Replication		Primary		Replication	
	N = 77		N = 35		N = 31		N = 48		N = 31	
**Female (%)**	43		46		55		29		42	
	**Mean**	**SD**	**Mean**	**SD**	**Mean**	**SD**	**Mean**	**SD**	**Mean**	**SD**
**Gestational Age**	275	15	281	10	282	14	277	15	277	12
**(days)**										
	**Cell-Type Proportions**									
**B cells**	0.08	0.03	0.08	0.03	0.08	0.02	0.08	0.03	0.08	0.02
**Granulocytes**	0.39	0.08	0.4	0.1	0.41	0.07	0.39	0.08	0.38	0.07
**Monocytes**	0.18	0.03	0.18	0.03	0.18	0.03	0.18	0.03	0.18	0.03
**Natural killer cells**	0.05	0.02	0.05	0.02	0.05	0.02	0.05	0.02	0.05	0.02
**Cytotoxic T cells**	0.15	0.04	0.15	0.04	0.14	0.04	0.15	0.04	0.16	0.04
**T-helper cells**	0.15	0.04	0.14	0.05	0.14	0.03	0.14	0.04	0.15	0.04

N is the number of participants in the primary and replication datasets, respectively, for each of the two treatments and for untreated individuals.

## Methylome-wide profiles and estimated cell-type proportions

As recently described [20], methylome-wide profiles from each neonatal blood sample were generated using an optimized procedure for methyl-binding domain enrichment sequencing (MBD-seq), a sequencing-based technique that enables the detection of nearly all 28 million CpGs in the human genome [29–31]. After quality control, e.g., removing methylation sites that were unmethylated in nearly all participants, this dataset [20] involved high quality methylation profiles consisting of, on average, 46.2 million (SD = 5.9) reads for each of the 222 included participants, that investigated 24,244,667 common CpG methylation sites. Cell-type proportions were estimated directly from the methylation data from each sample, using a recently generated neonatal reference panel of DNAm profiles from purified neonatal blood cell-types consisting of the most common blood cell-types in neonatal samples: B cells, granulocytes, monocytes, natural killer (NK) cells, cytotoxic T cells and T-helper cells [20]. Due to the design of the reference panel no further subsets of cell-types were investigated.

### Methylome-wide association study (MWAS)

Methylome-wide association studies (MWAS) were performed using RaMWAS, a Bioconductor package specifically designed for large-scale DNA methylation studies [32] that allows for statistical testing using linear/logistic regression while controlling for covariates. Covariates included in the analysis were possible assay related artifacts such as the efficiency of the methylation enrichment procedure, percentage reads aligned, and sample batch; as well as participant specific variables such as sex, birth year, gestational age and cell-type proportions. After regressing out measured covariates, we performed a principal component analysis on the methylation data. Based on a scree plot, we identified two principal components, which captured any remaining unmeasured confounders, to be included in the analyses as covariates.

In addition to performing MWAS of whole blood, we used a reference-based statistical deconvolution approach that has been carefully described and evaluated previously [33] to perform association testing on a cell-type specific level. With this approach, the cell-type proportions in combination with the statistical deconvolution algorithm are applied to disentangle the association signal for each cell-type [33, 34]. A full description is provided in the Supporting information.

To account for multiple testing and declare methylome-wide significance, we controlled the false discovery rate (FDR) at the 0.1 level, (i.e., q<0.1). Thus, on average 10% of the sites declared significant are expected to be false discoveries, which provides a good balance between finding true associations and avoiding false discoveries [35]. Suggestively significance was defined as a p-value (p) < 1.0x10^-6^.

### Replication of MWAS findings

To evaluate the robustness of the MWAS results we also performed replication, where fully independent treated individuals were compared against the same untreated participants as in the primary analyses, using the same approach as for the primary analyses. Specifically, we performed "look-up replication” of all CpGs in the corresponding cell-type that achieved methylome-wide significance in the primary dataset. To control for multiple testing, we applied Bonferroni correction for the total number of CpGs tested, across the cell-types, in the replication phase.

Furthermore, to evaluate the overall robustness of the MWAS results we performed enrichment testing, where the suggestively significant (p<1x10^-6^) MWAS results from the primary analyses were tested for overlap with nominally significant (p<0.05) findings from the replication data. The approach uses a permutation-based enrichment test described previously [36], where the CpGs of the two data sets are mapped to each other based on chromosomal location. Next, 10,000 circular permutations [37] performed with the R-package shiftR was used to generate the empirical test statistic distribution under the null hypothesis assuming no enrichment. These permutations shift the mapping of the two data sets by a random number to preserve the correlational structure of the test statistics, which allows control for type I errors even amongst highly correlated sites [37].

### Testing for enrichment of overlap between treatments

To test for similarity in associated methylation marks across the two treatments, we again used permutation-based enrichment analysis, described above, to examine whether the suggestively significant MWAS findings from one treatment were enriched for top findings from the other treatment (laughing gas vs. pudendal block), and vice versa. To focus our analyses on overlap among suggestively significant findings, we used a threshold of p<1x10^-6^ for both the primary dataset, and for the replication dataset.

### Gene Ontology analyses

Using the primary MWAS results, we performed permutation-based enrichment analyses of Gene Ontology (GO) terms from level 5, using data from ConsensusPathDB-human release 35 (http://cpdb.molgen.mpg.de/) [38]. For each outcome variable, we performed the permutation test with all genes linked to suggestively significant (p < 1.0x10^-6^) MWAS findings detected across cell-types and bulk, for each treatment. A gene was considered linked if the CpG site was located within the body of the gene or within 10,000 base pairs upstream of the transcription start site, aimed at accounting for methylation sites in promoter regions. The analyses were performed with the shiftR R-package, and 10,000 circular permutations as described above. Significant GO terms (p < 0.05) with at least three overlapping genes were then clustered based on gene overlap, using the Louvain Method for community detection for pathway clustering [39].

## Results

### Primary MWAS results

Our primary MWAS analysis includes participants whose mothers received exclusive treatment of either laughing gas or pudendal block, as well as a control group of individuals whose mothers did not receive any pain relief. For each of the two treatment groups versus controls, we performed MWAS of whole blood and for each of six common cell-types in neonatal blood (B cells, granulocytes, monocytes, natural killer (NK) cells, cytotoxic T (Tc) cells, and T-helper (Th) cells). S1 Fig in S1 File shows quantile-quantile plots and corresponding lambdas (i.e., the ratio of the median observed test-statistic distribution to the expected under the null hypothesis) for each of the MWAS. The shape of these plots and the range of the lambdas (0.993–1.095) show no indications of test statistic inflation, supporting the accuracy of our p values.

Our results identified two methylome-wide significantly associated (q < 0.1) findings for laughing gas (Table 2) and five for pudendal block. More specifically, a methylome-wide significant locus for laughing gas, involving two CpGs, was detected in granulocytes (p < 5.50 x10^-9^, q = 0.067) on chromosome 3q26.1, in an intergenic region. For pudendal block, a methylome-wide significant loci involving two CpGs, was detected for monocytes (p < 1.51 x10^-8^, q = 0.073) on chromosome 2q36, which is directly overlapping with the *Sphingosine—phosphate phosphatase 2 (SGPP2)* gene. Two additional loci, involving 2 CpGs on chromosome 4q26 (p < 1.47 x10^-8^, q = 0.073) and a single CpG on 11p15.4 (p = 1.51 x10^-8^, q = 0.073), were detected for monocytes. Both loci were located in intergenic regions.

**Table 2:** Methylome-wide significant findings (q < 0.1) for laughing gas and pudendal block in primary MWAS and follow-up with look-up replication.

Cell Type	Chr.	Location (bp)	Gene Name	Primary MWAS			Look-up Replication	
				Partial Correlation	p value	q value	Partial Correlation	p value
**Laughing gas**								
Granulocytes	3	162,317,671		0.59	4.40E-09	0.067	0.36	1.03E-03
Granulocytes	3	162,317,679		0.59	5.50E-09	0.067	0.33	3.06E-03
**Pudendal block**								
Monocytes	2	223,333,925	SGPP2	0.55	7.91E-09	0.073	0.35	1.44E-03
Monocytes	2	223,333,933	SGPP2	0.54	1.51E-08	0.073	0.34	2.38E-03
Monocytes	4	114,685,189		0.54	1.47E-08	0.073	0.47	1.19E-05
Monocytes	4	114,685,229		0.55	6.94E-09	0.073	0.50	2.89E-06
Monocytes	11	5,166,625		0.54	1.51E-08	0.073	-	9.70E-02

Abbreviations—Chr, chromosome: bp, base pair. Partial correlation not reported for non-significant results.

Furthermore, we identified suggestively significant (p<1x10^-6^) methylation marks for both laughing gas (N = 267 CpGs) and for pudendal block (N = 288 CpGs) (S1 and S2 Tables). When comparing the results for the two treatments, for each cell-type, no suggestively significant findings from one treatment was detected in the other.

### Replication of MWAS results

A look-up replication of the methylome-wide significant CpGs in the replication dataset confirmed significant findings in three of the four loci (Table 2, right side). For each of the three loci, both CpGs replicated following Bonferroni correction for seven tests. The single CpG detected on chromosome 11, for monocytes, did not replicate.

Furthermore, we replicated our suggestively significant primary MWAS results using a permutation-based enrichment test. The results showed significant enrichment of nominally significant findings in the replication sample for all cell-types and bulk, for both laughing gas (odds ratios (OR) ranging 4.53 to 39.02; p<4.00×10^−4^) and pudendal block (OR ranging 3.47 to 25.93, p<3.00×10^−4^), supporting robustness of our findings (S3 Table in S1 File).

### Gene Ontology analyses of MWAS findings

**Fig 1:**
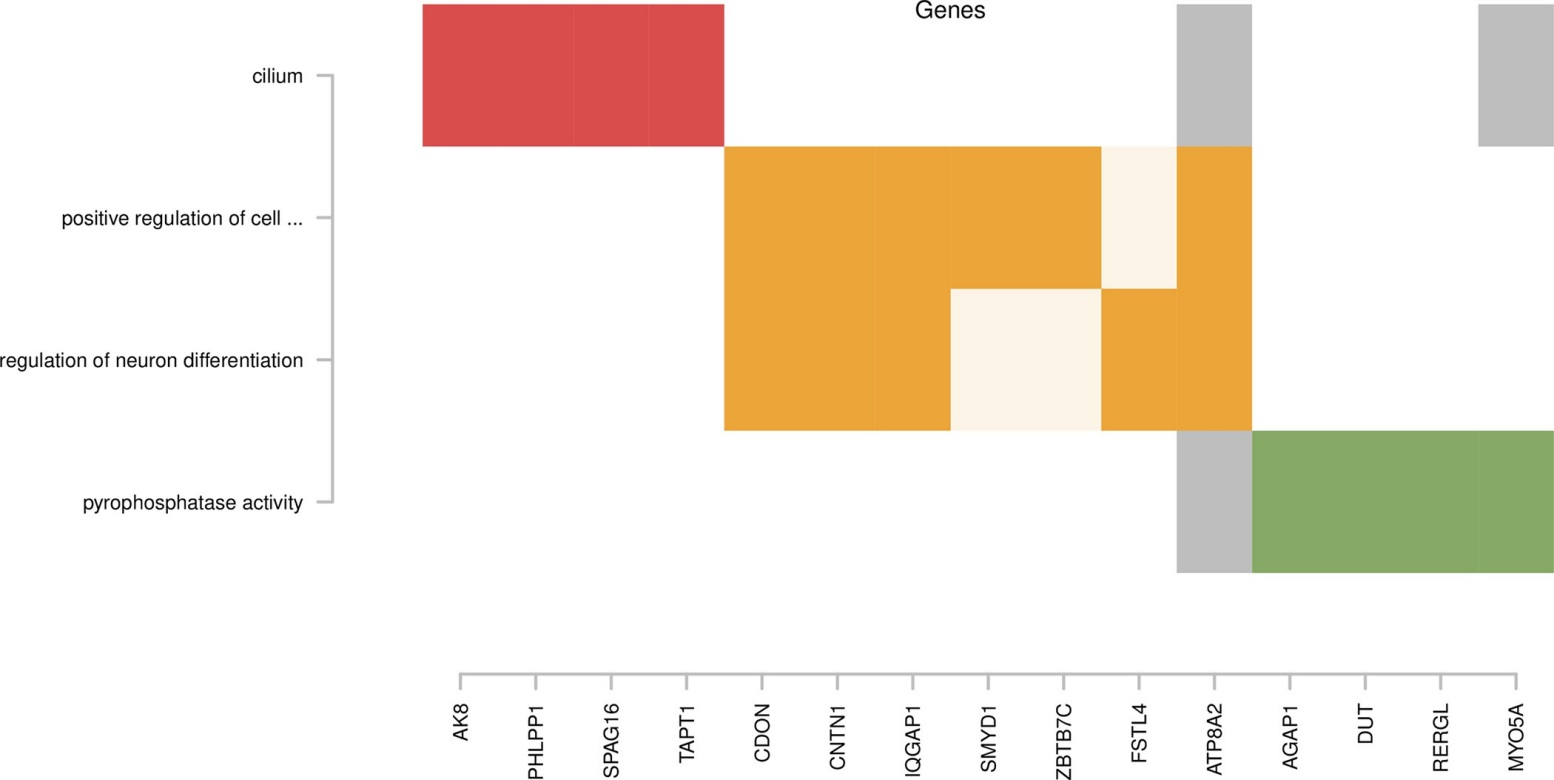
Clusters of significant Gene Ontology terms for laughing gas treatment. The X-axis indicates genes linked to suggestively significant associations and the Y-axis shows significant Gene Ontology terms detected. Colored boxes indicate defining genes of clustering terms, corresponding to the colors indicated in Table 3. Grey boxes indicate genes also present in terms assigned to other clusters where they are not considered defining.

Gene ontology analyses were performed using genes linked to suggestively significant findings from the primary MWAS that overlapped with sites from the replication data, i.e., only sites that contributed to the gene overlap were further investigated. For laughing gas, we identified four significant GO terms (Table 3, top), with up to six genes each, that formed three clusters (Fig 1). The most significant term was related to cilium (GO:0005929, OR = 6.00, p = 0.0014). The largest cluster (yellow), i.e., the only cluster that contained more than one term, was related to regulation of cell differentiation, and included two terms, positive regulation of cell differentiation (GO:0045597, OR = 3.44, p = 0.0235) and regulation of neuron differentiation (GO:0045664, OR = 4.28, p = 0.0373).

**Fig 2:**
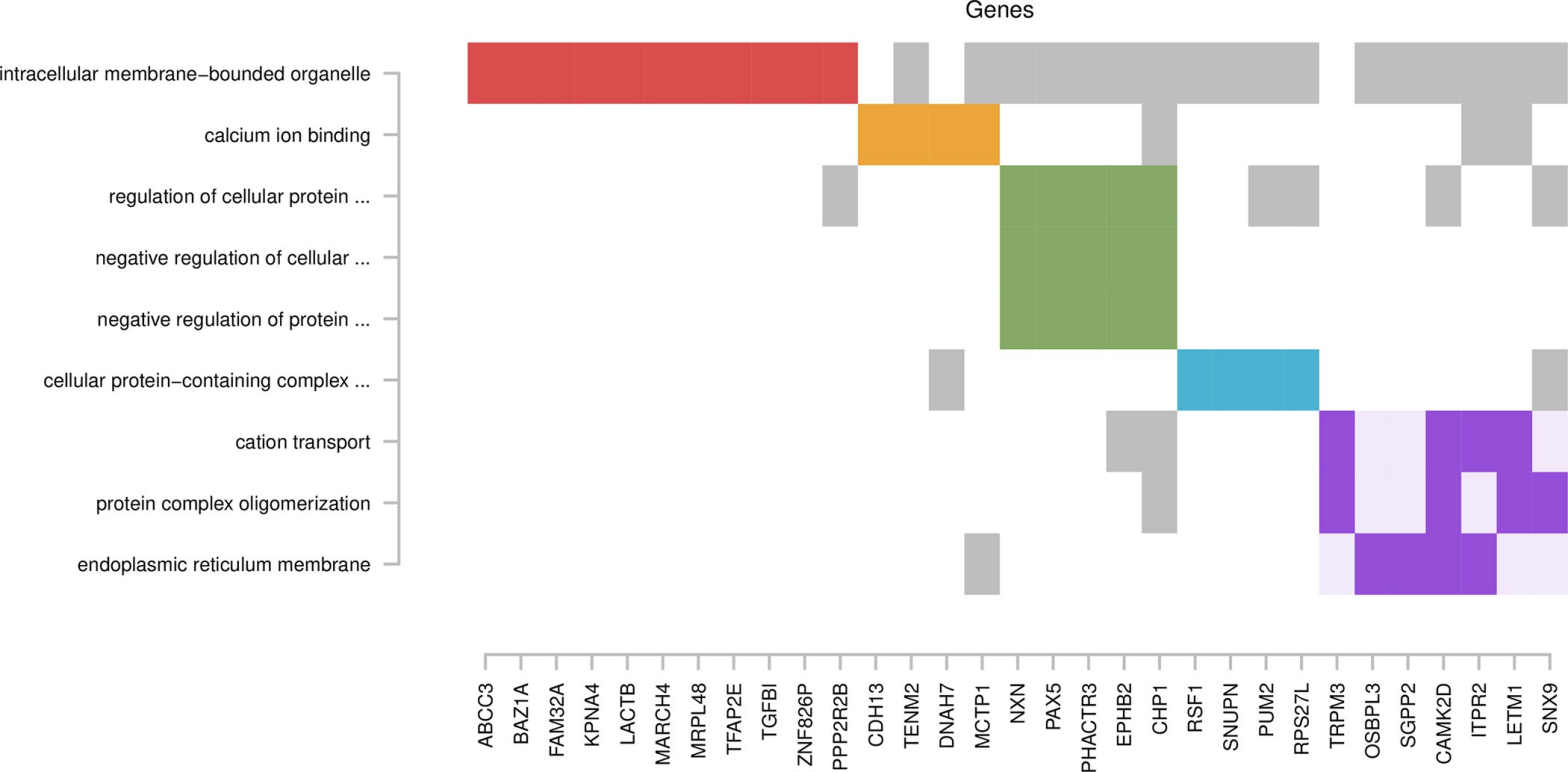
Clusters of significant Gene Ontology terms for pudendal block treatment. The X-axis indicates genes linked to suggestively significant associations and the Y-axis shows significant Gene Ontology terms detected. Colored boxes indicate defining genes of clustering terms, corresponding to the colors indicated in Table 3. Grey boxes indicate genes also present in terms assigned to other clusters where they are not considered defining.

**Table 3:** GO terms detected for laughing gas and pudendal block.

Cluster	GO ID	GO term	Overlapping genes	Odds ratio	p value
**Laughing Gas**					
Red	0005929^c^	cilium	6	6.00	0.0014
Yellow	0045597	positive reg of cell differentiation	6	3.44	0.0235
Yellow	0045664	reg of neuron differentiation	5	4.28	0.0373
Green	0016462^f^	pyrophosphatase activity	5	2.88	0.0309
**Pudendal Block**					
Red	0043231^c^	intracellular membrane-bounded organelle	28	1.83	0.0458
Yellow	0005509^f^	calcium ion binding	7	5.50	0.0179
Green	0032268	reg of cellular protein metabolic process	10	2.14	0.0331
Green	0032269	negative reg of cellular protein metabolic process	5	2.53	0.0333
Green	0051248	negative reg of protein metabolic process	5	2.37	0.0447
Blue	0034622	cellular protein-containing complex assembly	6	2.85	0.0135
Purple	0006812	cation transport	6	2.79	0.0430
Purple	0051259	protein complex oligomerization	5	4.93	0.0039
Purple	0005789^c^	endoplasmic reticulum membrane	5	2.41	0.0287

Cluster indicates corresponding cluster in Figs 1 and 2 for laughing gas and pudendal block, respectively. Unless otherwise specified, GO terms are part of the biological processes category. Superscript ^f^ and ^c^ denote terms that belong to the biological function or cellular component categories, respectively.

For pudendal block, we identified nine significant GO terms (Table 3, bottom), including up to 28 genes each, that formed five clusters (Fig 2). One of two multi-term clusters (green) involved three terms related to regulation of metabolic processes. The largest gene overlap for this cluster was detected for regulation of cellular protein metabolic process (GO:0032268, OR = 2.14, p = 0.0331). Furthermore, the 28 genes largest gene overlap overall involved and was observed for intracellular membrane-bound organelles (GO:0043231, OR = 1.83, p = 0.0458). Ten of the 28 genes were not observed for any other enriched term and therefore this term did not cluster with other terms. Another single-term cluster (yellow) involved calcium ion binding (GO:0005509, OR = 5.50, p = 0.0179). This term involved seven overlapping genes, two of which were not part of the large red cluster. The only other cluster that involved genes that was not part of the intracellular membrane-bound organelles term was a functionally diverse cluster (purple) involving three terms. This cluster also involved the most significant term for pudendal block overall, a term related to protein complex oligomerization (GO:0051259, OR = 4.93, p = 0.0039).

## Discussion

To our knowledge, this is the first methylome-wide investigation of the effects of maternal obstetric pain relief on the neonatal blood methylome. In addition to studying the potential effects of these treatments in whole blood we performed cell-type specific analyses that allowed us to identify methylation differences occurring in specific cell-types. Our MWAS analyses detected methylome-wide significant methylation marks in the newborn for laughing gas in granulocytes and for pudendal block in monocytes, the majority of which replicated in different individuals that received similar treatment. Suggestively significant findings were detected in bulk and in all cell-types, for both treatments. Further replication analyses imply that our suggestively significant results were robust, with significant enrichment of replicating findings for both treatments, for bulk and in all cell-types. Interestingly, when comparing suggestively significant findings across treatments, our results suggest that the two treatments are associated with different alterations to the neonatal blood methylome, and that these alterations are involved in different biological functions.

The methylome-wide significant finding for laughing gas in granulocytes was located in an intergenic region on chromosome 3, overlapping a repeating element, with unknown function. However, although not strictly considered methylome-wide significant, the second most significant finding for laughing gas (p = 4.77x10^-9^, q = 0.116, S1 Table), detected in natural killer cells, was linked to the *CDON* gene. This gene is included in both GO terms of the yellow cluster (Fig 1) where it is involved in cell differentiation. The protein levels of this gene have previously been reported as decreased in plasma proteome investigations following fasting and general anesthesia [40], opening for the possibility that this gene is also responsive to obstetric pain relief that is detectable in the neonate.

Similar to the methylome-wide significant finding for laughing gas, the replicating methylome-wide significant finding for pudendal block in monocytes on chromosome 4 was located in an intergenic region. In contrast the replicating loci on chromosome 2 overlapped the *SGPP2* gene. This gene is involved in the breakdown of sphingosine-1-phosphate (S1P), a bioactive sphingolipid molecule of importance for many physiological processes, such as, cell signaling, angiogenesis, immune system regulation and vascular tone. GO analyses of replicating suggestively significant findings (Fig 2), places *SGPP2* in a significant term, with the largest overlapping set of genes detected, of importance for the intracellular membrane-bound organelle, i.e., a general function for the cell’s viability and communication ability. In addition, the analyses place *SGPP2* in a second significant GO term related specifically to the endoplasmic reticulum, which is a critical organelle.

As shown in Fig 2, there are only three overlapping genes that were detected for significant GO terms that were not part of the intracellular membrane-bound organelle term (red). Two of these genes were included in the calcium ion binding term (yellow) and the third was included in, for example, cation transport (purple), which includes the transport of calcium ions, suggesting that calcium related functions are of relevance for the associations detected for pudendal block. Interestingly, calcium plays a critical role in many aspects of pain pathophysiology [41] and has previously been studied in relation to anesthesia [42, 43].

We did not observe overlap in suggestive results between laughing gas and pudendal block nor did we observe the same GO terms for the two treatment types. This absence of overlap suggests treatment-specific alterations to the methylome, indicating that the impacts of laughing gas and pudendal block are discernibly unique in terms of their epigenetic effects and accentuates the likely specificity of the molecular responses induced by each treatment. On a more technical level, this lack of overlap between the two treatment types also indicated that the use of the same controls for the two treatments, and for the primary phase as well as for the replication phase, do not have a major confounding effect on suggestively significant findings.

In this study, we provide novel information about alterations in the neonatal blood methylome that potentially occur as a result of maternal pain relief during delivery. While this information may contribute to an improved understanding of the biological functions and consequences related to these treatments, it is important to acknowledge potential limitations when interpreting the results. The investigated samples originate from a relatively homogenous cohort, comprising individuals of Swedish descent whose information was collected via a standardized national hospital system. This homogeneity may potentially limit the generalizability of the results towards more diverse populations. Furthermore, the study sample was originally collected to investigate schizophrenia, and therefore includes individuals that later in life, typically in late adolescence or early adulthood, developed this disorder. Therefore, to evaluate the potential effect of schizophrenia on our results, we investigated the correlation between treatment and schizophrenia diagnosis later in life. No significant correlation was observed (Person’s correlation = 0.01/0.03, p = 0.83/0.69 for laughing gas and pudendal block, respectively). Thus, as schizophrenia case-control status is uncorrelated with treatment group, it should not be considered a confounding factor for the observed effects. Moreover, the investigated treatment groups are relatively small, leading to limited power to detect alterations, yet we detect highly significant and replicating robust findings. Thus, it is possible, and even likely, that influenced methylation marks may go undetected. However, it is important to note that the limited power does not influence the validity of the detected findings. Along the same lines, the treated individuals in the primary samples received only one treatment. However, replication samples received either of the two treatments and at least one additional treatment. This combination treatment may dilute or alter the effects of laughing gas or pudendal block, possibly leading to that some influenced methylation marks going undetected in the replication sample. Finally, it is important to note that the effects of the detected methylation alterations and their potential disadvantageous, or advantageous, influences on the child’s future health and development remains to be determined.

In conclusion, our cell-type specific methylome-wide investigation is the first to show the effect of obstetric pain-relief on the blood methylome in the newborn child. The observed differences suggest that anesthetic treatment, such as laughing gas or pudendal block, administered to the mother during delivery may alter the neonatal methylome in a cell-type specific manner. Some of the detected findings are involved in biological functions that previously have been investigated in relation to anesthetic treatment, supporting its potential role also in obstetric anesthesia.

## Supporting information

S1 File Supporting information. Methods, S1 Fig, S3 Table.
(PDF)

S1 Table Laughing gas bulk and cell-type MWAS top results. (XLSX)

S2 Table Pudendal block bulk and cell-type MWAS top results. (XLSX)
