# Supplementary material for: Cell-type specific methylation changes in the newborn child associated to obstetric pain relief
Source: PLoS One. 2024 Sep 19;19(9):e0308644. doi: 10.1371/journal.pone.0308644 (PMC11412656; doi:10.1371/journal.pone.0308644)
Supplement: S1 File — Methods, S1 Fig, S3 Table. (PDF) [file pone.0308644.s001.pdf]

Supporting Information for:

Cell-type specific methylation changes in the newborn child associated to obstetric pain relief

TABLE OF CONTENT

METHODS 3

CELL-TYPE SPECIFIC METHYLOME-WIDE ASSOCIATION STUDIES (MWASS) 3

FIGURES 5

S1 FIG. QQ PLOTS FOR METHYLOME-WIDE ASSOCIATIONS STUDIES 5

TABLES 7

S1 TABLE. LAUGHING GAS BULK AND CELL-TYPE MWAS TOP RESULTS 7

S2 TABLE. PUDENDAL BLOCK BULK AND CELL-TYPE MWAS TOP RESULTS 7

THE FULL MWAS RESULTS ARE AVAILABLE VIA S4-S17 FILE IN RDS FORMAT. 7

S3 TABLE. ENRICHMENT TESTING OF PRIMARY MWAS VS. REPLICATION MWAS 7

REFERENCES 8

Methods

Cell-type specific methylome-wide association studies (MWASs)

Cell-type proportions were estimated directly from the methylation data from each sample, using an empirical Bayes estimator[1]. This approach uses a recently created MBD-seq based neonatal reference panel that includes profiles from the most common neonatal blood cell-types in neonates: B cells, granulocytes, monocytes, natural killer (NK) cells, cytotoxic T (Tc) cells, and T-helper (Th) cells[2].

The cell-type specific MWASs were performed using a statistical deconvolution approach that has been carefully described and evaluated previously[3]. Statistical deconvolution, which was introduced 20 years ago, is widely used in gene expression studies[1, 4, 5] and has been applied to DNA methylation studies by us and others[2, 6-8]. In short, the cell-type proportions in combination with the statistical deconvolution algorithm are applied to disentangle the association signal for each cell-type[3, 9]. The statistical model for the cell-type specific analyses is:

$$Y^{bulk} = \sum_{c=1}^{n_c} m_c P_c + \sum_{c=1}^{n_c} m_c^{NO}(NO \times P_c) + E$$

Thus, measurements from bulk tissue Y^{bulk} are regressed on $c = 1$ to n_c, cell-type proportions P_c, and the product of the neonatal outcome (NO) by cell-type proportions $(NO \times P_c)$. The model allows for covariates (not shown) and residual effects E. Coefficient m_c is the effect of cell-type c. The case-control difference m_c^{NO} for cell-type c is used to test the null hypothesis that cell-type means are equal for cases

and controls. QQ plots and lambda, calculated as the median of the observed results divided by the expected median of the null distribution. were evaluated for signs of test statistic inflation (S1 Fig).

Figures

S1 Fig. QQ plots for methylome-wide associations studies

A. Laughing Gas

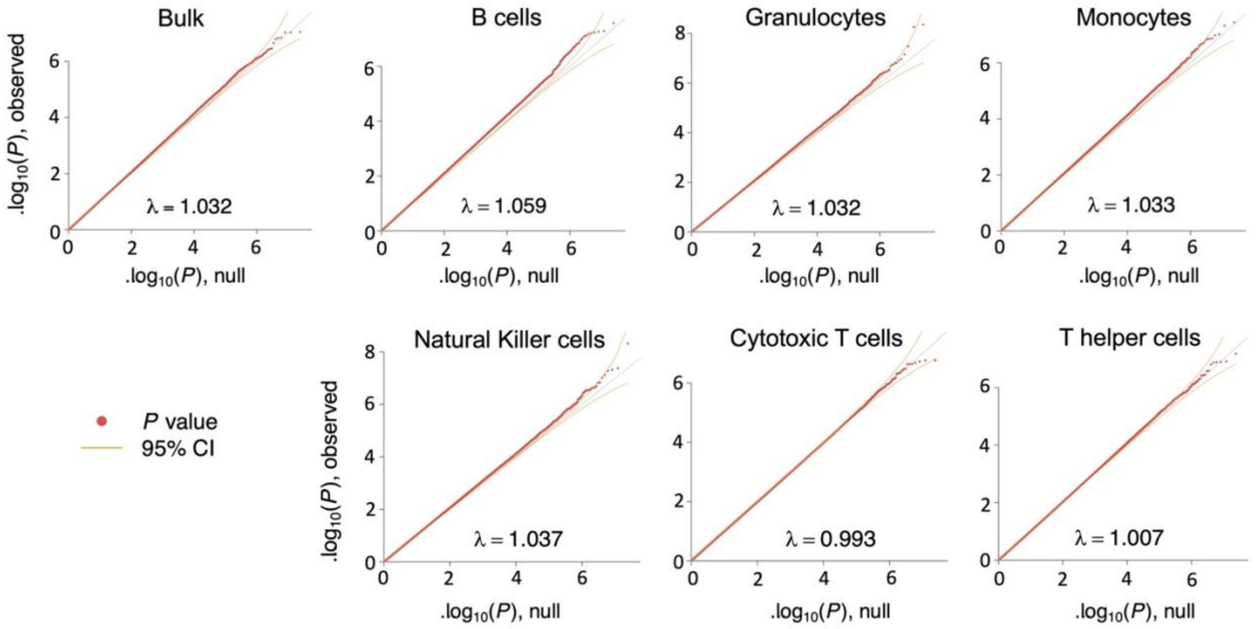

B. Pudendal Block

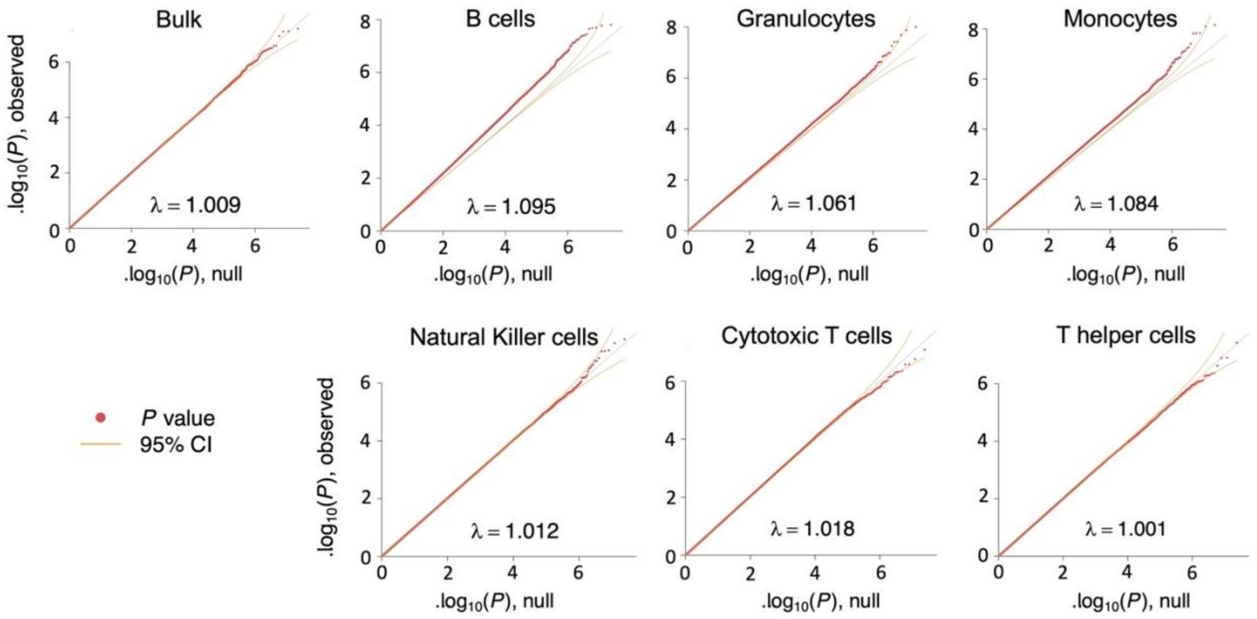

QQ plots and lambda for Methylome-wide Associations Studies for bulk and six cell-types. Y-axis shows the $log_{10}(P)$ of the observed data and the X-axis displays the $log_{10}(P)$ of the null.

Tables

S1 Table. Laughing gas bulk and cell-type MWAS top results

See separate excel file: S1 Table Laughing gas bulk and cell-type MWAS top results

S2 Table. Pudendal block bulk and cell-type MWAS top results

See separate excel file: S2 Table Pudendal block bulk and cell-type MWAS top results

S3 Table. Enrichment testing of primary MWAS vs. replication MWAS

Cell-type	Enrichment Ratio	p value
	**Laughing Gas**	-
Bulk	13.39	<1.00E-04
B cells	4.53	4.00E-04
Granulocytes	8.09	<1.00E-04
Monocytes	9.05	<1.00E-04
Natural Killer cells	6.80	<1.00E-04
Cytotoxic T cells	18.23	<1.00E-04
T Helper cells	39.02	<1.00E-04
	**Pudendal Block**	
Bulk	17.57	<1.00E-04
B cells	3.47	3.00E-04
Granulocytes	9.18	<1.00E-04
Monocytes	12.98	<1.00E-04
Natural Killer cells	38.70	<1.00E-04
Cytotoxic T cells	12.31	2.00E-04
T Helper cells	25.93	<1.00E-04

References

1. van den Oord EJCG, Aberg KA. Fine-grained cell-type specific association studies with human bulk brain data using a large single-nucleus RNA sequencing based reference panel. Scientific Reports. 2023;13(1):13004. doi: 10.1038/s41598-023-39864-2.

2. van den Oord E, Xie LY, Zhao M, Campbell TL, Turecki G, Kahler AK, et al. Genes implicated by a methylome-wide schizophrenia study in neonatal blood show differential expression in adult brain samples. Mol Psychiatry. 2023;28(5):2088-94. Epub 20230427. doi: 10.1038/s41380-023-02080-5. PubMed PMID: 37106120.

3. Venet D, Pecasse F, Maenhaut C, Bersini H. Separation of samples into their constituents using gene expression data. Bioinformatics. 2001;17 Suppl 1:S279-87. Epub 2001/07/27. doi: 10.1093/bioinformatics/17.suppl_1.s279. PubMed PMID: 11473019.

4. Guintivano J, Aberg KA, Clark SL, Rubinow DR, Sullivan PF, Meltzer-Brody S, et al. Transcriptome-wide association study for postpartum depression implicates altered B-cell activation and insulin resistance. Molecular Psychiatry. 2022;27(6):2858-67. doi: 10.1038/s41380-022-01525-7.

5. Shen-Orr SS, Tibshirani R, Khatri P, Bodian DL, Staedtler F, Perry NM, et al. Cell type-specific gene expression differences in complex tissues. Nat Methods. 2010;7(4):287-9. Epub 2010/03/09. doi: nmeth.1439 [pii]
10.1038/nmeth.1439. PubMed PMID: 20208531; PubMed Central PMCID: PMC3699332.

6. Chan RF, Turecki G, Shabalin AA, Guintivano J, Zhao M, Xie LY, et al. Cell Type-Specific Methylome-wide Association Studies Implicate Neurotrophin and Innate Immune Signaling in Major Depressive Disorder. Biol Psychiatry. 2020;87(5):431-42. doi: 10.1016/j.biopsych.2019.10.014. PubMed PMID: 31889537.

7. Zheng SC, Breeze CE, Beck S, Teschendorff AE. Identification of differentially methylated cell types in epigenome-wide association studies. Nat Methods. 2018;15(12):1059-
8

66. doi: 10.1038/s41592-018-0213-x. PubMed PMID: 30504870; PubMed Central PMCID: PMCPMC6277016.

8. Titus AJ, Gallimore RM, Salas LA, Christensen BC. Cell-type deconvolution from DNA methylation: a review of recent applications. Hum Mol Genet. 2017;26(R2):R216-R24. doi: 10.1093/hmg/ddx275. PubMed PMID: 28977446; PubMed Central PMCID: PMCPMC5886462.

9. Wang X, Park J, Susztak K, Zhang NR, Li M. Bulk tissue cell type deconvolution with multi-subject single-cell expression reference. Nat Commun. 2019;10(1):380. Epub 20190122. doi: 10.1038/s41467-018-08023-x. PubMed PMID: 30670690; PubMed Central PMCID: PMCPMC6342984.
